# System-wide coordinates of higher order functions in host-pathogen environment upon *Mycobacterium tuberculosis* infection

**DOI:** 10.1038/s41598-018-22884-8

**Published:** 2018-03-22

**Authors:** P. V. Parvati Sai Arun, Sravan Kumar Miryala, Aarti Rana, Sreenivasulu Kurukuti, Yusuf Akhter, Sailu Yellaboina

**Affiliations:** 1 C R Rao AIMSCS, University of Hyderabad campus, Hyderabad, Telangana India; 2 IOB-YU Centre for Systems Biology and Molecular Medicine, Yenepoya Research Centre Yenepoya University, Mangalore, Karnataka India; 3 Centre for Computational Biology and Bioinformatics, School of Life Sciences, Central University of Himachal Pradesh, Dharamshala, India; 4 Department of Animal Biology, School of Life Sciences, University of Hyderabad, Hyderabad, India; 5 Department of Biotechnology, Babasaheb Bhimrao Ambedkar University, Vidya Vihar, Raebareli Road, Lucknow, Uttar Pradesh 226025 India

## Abstract

Molecular signatures and their interactions behind the successful establishment of infection of *Mycobacterium tuberculosis* (*Mtb*) inside macrophage are largely unknown. In this work, we present an inter-system scale atlas of the gene expression signatures, their interactions and higher order gene functions of macrophage-*Mtb* environment at the time of infection. We have carried out large-scale meta-analysis of previously published gene expression microarray studies andhave identified a ranked list of differentially expressed genes and their higher order functions in intracellular *Mtb* as well as the infected macrophage. Comparative analysis of gene expression signatures of intracellular *Mtb* with the *in vitro* dormant *Mtb* at different hypoxic and oxidative stress conditions led to the identification of the large number of *Mtb* functional groups, namely operons, regulons and pathways that were common and unique to the intracellular environment and dormancy state. Some of the functions that are specific to intracellular *Mtb* are cholesterol degradation and biosynthesis of immunomodulatory phenolic compounds. The molecular signatures we have identified to be involved in adaptation to different stress conditions in macrophage environment may be critical for designing therapeutic interventions against tuberculosis. And, our approach may be broadly applicable for investigating other host-pathogen interactions.

## Introduction

*Mycobacterium tuberculosis* (*Mtb*) is the causative factor for tuberculosis (TB). Besides HIV, *Mtb* is the leading cause of death worldwide^1–3^. After successful infection into its host, Mtb survives as an intracellular pathogen, localized inside the macrophages and dendritic cells^4,5^. The phenomenon of infection of a healthy macrophage by *Mtb* can be divided into three inter-related stages such as triggering inflammatory response from macrophage upon infection; activation of cell mediated immunity and followed by formation of granulomas, and finally the reactivation of *Mtb* due to various environmental and genetic factors^6^. *Mtb* is known to survive for many years under the adverse intracellular environment of macrophages by down regulating the metabolic pathways and switching to non-replicative state^7,8^. It was observed that to survive in the adverse conditions, *Mtb* not only alters its gene expression profiles but also it alters the gene expression profiles of the host^9^.

Previously, many independent microarray studies were conducted to identify differentially expressed genes in *Mtb* as well as macrophages during the stable infection^10–19^. In addition to these studies, there are also few gene expression microarray studies conducted to identify adaptive mechanisms of *Mtb* to adverse conditions in macrophages by using *in-vitro* models such as nutrient starvation, hypoxia, and oxidative stress^20,21^. Furthermore, Meta-analysis of gene expression microarray data from independent and related datasets was also performed in several studies by which the random errors created in experiments were nullified and also the right estimate of differential expression was obtained^22,23^. Moreover, by performing the meta-analysis, it was also found that there is an increase in the reliability and generalization of results^23–25^. In this work, we performed simultaneous meta-analysis of gene expression microarray data from infected host cell (macrophage) as well as pathogen (*Mtb*) to identify the ranked list of gene expression signatures induced in host-pathogen environment.

Furthermore, functional over-representation analysis (FOA) of different gene groups namely complexes, pathways, regulons and gene ontology terms among the gene expression signatures was also performed which led us to identify the system-wide functions in Mtb-macrophage environment. Inclusion of operons for FOA of Mtb gene expression signatures identified many unknown pathways/complexes and adaptive mechanisms with in the macrophage environment.

Furthermore, functional over-representation analysis (FOA) of different gene groups namely complexes, pathways, regulons and gene ontology terms among the gene expression signatures was also performed which led us to identify the system-wide functions in *Mtb*-macrophage environment. Inclusion of operons for FOA of *Mtb* gene expression signatures identified many unknown pathways/complexes and adaptive mechanisms within the macrophage environment.

## Materials and Methods

### Preparation of gene expression microarray datasets

Gene expression microarray data sets were downloaded from two major public repository databases Gene Expression Omnibus (GEO) (http://www.ncbi.nlm.nih.gov/geo/) and ArrayExpress (http://www.ebi.ac.uk/microarray-as/ae/). To remove the redundancy in the gene expression data, we have included the datasets from ArrayExpress database, if they are not found in GEO database. To facilitate the data comparison across the different platforms, gene expression microarray features were mapped to Entrez Gene IDS. Mapping information from gene names, refseq IDs, Ensembl IDs, Clone IDS, GenBank IDS to Entrez Gene IDS were obtained from various platform annotation files and also from various databases such as NCBI, BioMart, NIA array^26–28^.

### Differential expression analysis

In order to identify differentially expressed genes during infection, we considered the samples during the final stage of the infection. Differential expression analysis of gene expression microarray data was carried out using an R package called ‘RankProd’^29^. The package uses a modified ‘rank product’ method^29,30^ to identify up or down-regulated genes in one condition against another. We have used RankProd because, it uses Rank Product method which is a non-parametric statistical method that detects genes which are consistently found among the most strongly up-regulated genes in with replicate or without replicate experiments. It is based on the assumption that under the null hypothesis the order of all items is random. Probability of finding a specific item among the top of items in a list is k/n, where k is the rank of the item and n is the total number of items. Multiplying these probabilities gives the rank product. Smaller the rank product value, smaller the probability that the observed placement of the item at the top of the lists is due to chance.

### Meta-analysis of differential expression

Meta-analysis of gene expression microarrays was carried out using our previously described method^31^. We transformed the initial p-values of each gene obtained from the differential expression analysis of gene expression microarray datasets into the Z-scores, using the inverse cumulative distribution function (CDF)^32^. Then we took the weighted sum of the Z-scores and divided by the square root of sum of the weights to generate the average Z-score. Here the weights are equivalent to number of replicates in the gene expression microarray experiments. Finally the average Z-score was converted to p-value by using CDF^33^.

### Inference of interactions of between *Mtb* and Human

Experimentally verified Host-Pathogen interaction data from different bacterial species were retrieved from PHISTO and HPIDB databases^34,35^. PHISTO is a Pathogen Host Interaction Search Tool which contains a total of 23,633 pathogen host PPI data including 9318 interactions between bacteria and human. HPIDB is a Host Pathogen Interaction Database that currently contains 23.485 interactions between 66 host and 541 pathogen species. In addition, we curated a total of 53 experimentally verified host-pathogen protein-protein interaction information from 12 pathogens including *Mtb* from literature. The genome sequences of *Mtb* and other bacteria, which have experimentally verified interactions, were downloaded from NCBI ftp site (ftp://ftp.ncbi.nlm.nih.gov/genomes/). Then, we identified orthologs of *Mtb* in ~1500 sequenced genomes of bacterial species, which have experimentally verified host-pathogen interactions. Finally, the experimentally verified interactions were transferred to *Mtb* on the basis of orthology relationship. Totally we identified 2808 host-pathogen interactions between two sets of proteins 564 from Human and 1725 from *Mtb*.

### Preparation of functional gene groups

Annotated functions of human, i.e., pathways, motifs, gene ontology (GO) and immunological signatures were collected from Molecular Signature Database (MSigDB)^36,37^. Since the MSigDB does not maintain any functional gene groups for *Mtb*, we have curated the various functional gene groups such as complexes, pathways and operons etc., for *Mtb* from the published literature^38^.

### Functional over representation analysis of gene groups

Functional over-representation analysis (FOA) was carried out using our previously described method^39^. For each gene in a gene group, we have converted p-value of differential expression into Z-score using, inverse normal Cumulative Distribution Function (CDF). The FOA score of each gene group is the sum of the Z-scores divided by square root of number of genes. In order to identify the overrepresented target genes of various transcription regulators (regulon), we have taken sum of the absolute value of target genes Z-scores, then the total was divided by square root of number of genes. Finally the q-value was converted to P-value by using CDF. To calibrate the gene group (containing n genes) FOA score against the background distribution, we randomly sampled the n number of Z-scores and calculated the random FOA score. We repeated this process over 100,000 times and mean and standard deviation of the sampling distribution was used for correction of original score.

## Results and Discussion

Since both the host and the pathogen reciprocally influence the gene expression profiles of each other, it is pertinent to study the gene expression profiling of the pathogen as well as the host. This would provide insight into the strategies employed by the pathogen to survive in host environment and the host response to contest against the virulence of the pathogen^16^. Therefore, we performed the meta-analysis of gene expression changes in both *Mtb* and human macrophages during stable infection. In addition, we carried out Functional over-representation analysis (FOA) to identify higher order functions of induced or suppressed gene expression signatures both in *Mtb* as well as in the infected macrophages by considering functional groups of genes such as operons, regulons, pathways, complexes and biological process. Inclusion of diverse functional gene groups for the analysis ensured functional annotation for most of the genes. We extensively discuss the relevance of higher order functions of the genes in relation to the mechanisms adopted by the pathogen for its adaptation as well as the host defence.

### Meta-analysis of differentially expressed gene signatures during infection

**Figure 1:**
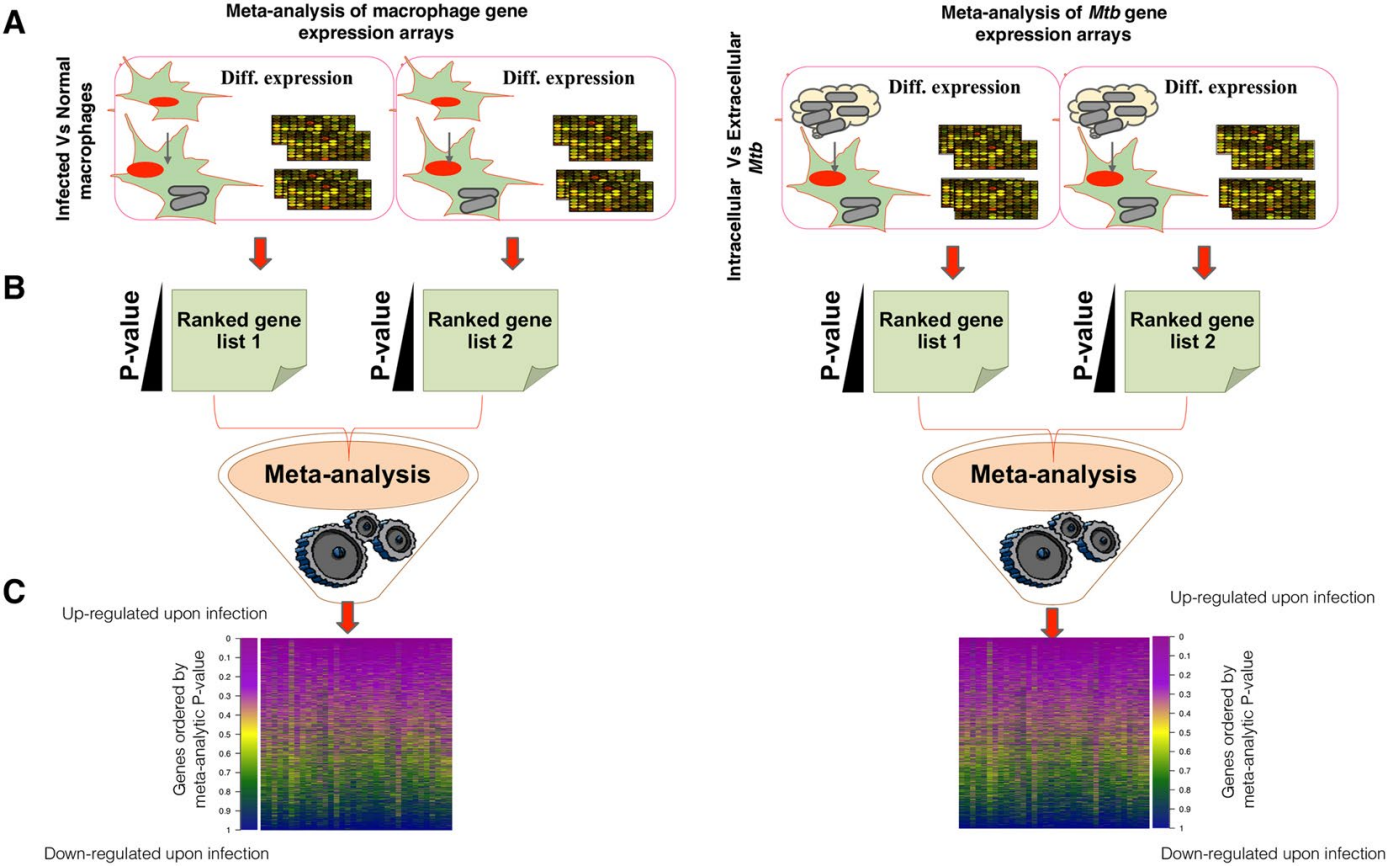
Meta-analytic approach to identify the gene expression signatures induced in host-pathogen environment upon infection by *Mtb*. (**A**) Initially the gene expression microarray datasets of human macrophages and *Mtb* were collected from Gene Expression Omnibus (GEO) (**B**). We have used R package to generate the ranked list of differentially expressed genes by comparing the samples of infected macrophages versus normal and *in vitro Mtb* vs. intracellular. (**C**) The ranked list of differentially expressed genes were combined using previously developed meta-analytic method to generate a consensus ranked list of the differentially expressed genes in macrophages as well as in *Mtb*.

We have developed a systematic frame work to identify the genes induced in host-pathogen environment (Fig. 1). Initially, we have identified two sets of differentially expressed genes, one from the *Mtb* and the other from the macrophages during the course of infection. Differentially expressed genes of *Mtb* were identified by comparing the gene expression data of *Mtb* which resides inside macrophage after infection (call it intracellular *Mtb*) with the *Mtb* before infection (*in vitro Mtb* cultures) using RankProd^29^. Similarly, we have also identified differentially expressed genes of macrophages by comparing gene expression data of the infected macrophages with the uninfected macrophages (Supplementary data S1). The differential gene expression scores were given ranks and then combined to generate a single consensus ranked list, using a meta-analytical method described previously^31^. The genes ranked atop the consensus ranked list are more severely and consistently up-regulated during infection across experiments, and those that are ranked at the bottom were down-regulated during infection.

### Host-pathogen interactions and essentiality of top ranked genes

**Figure 2:**
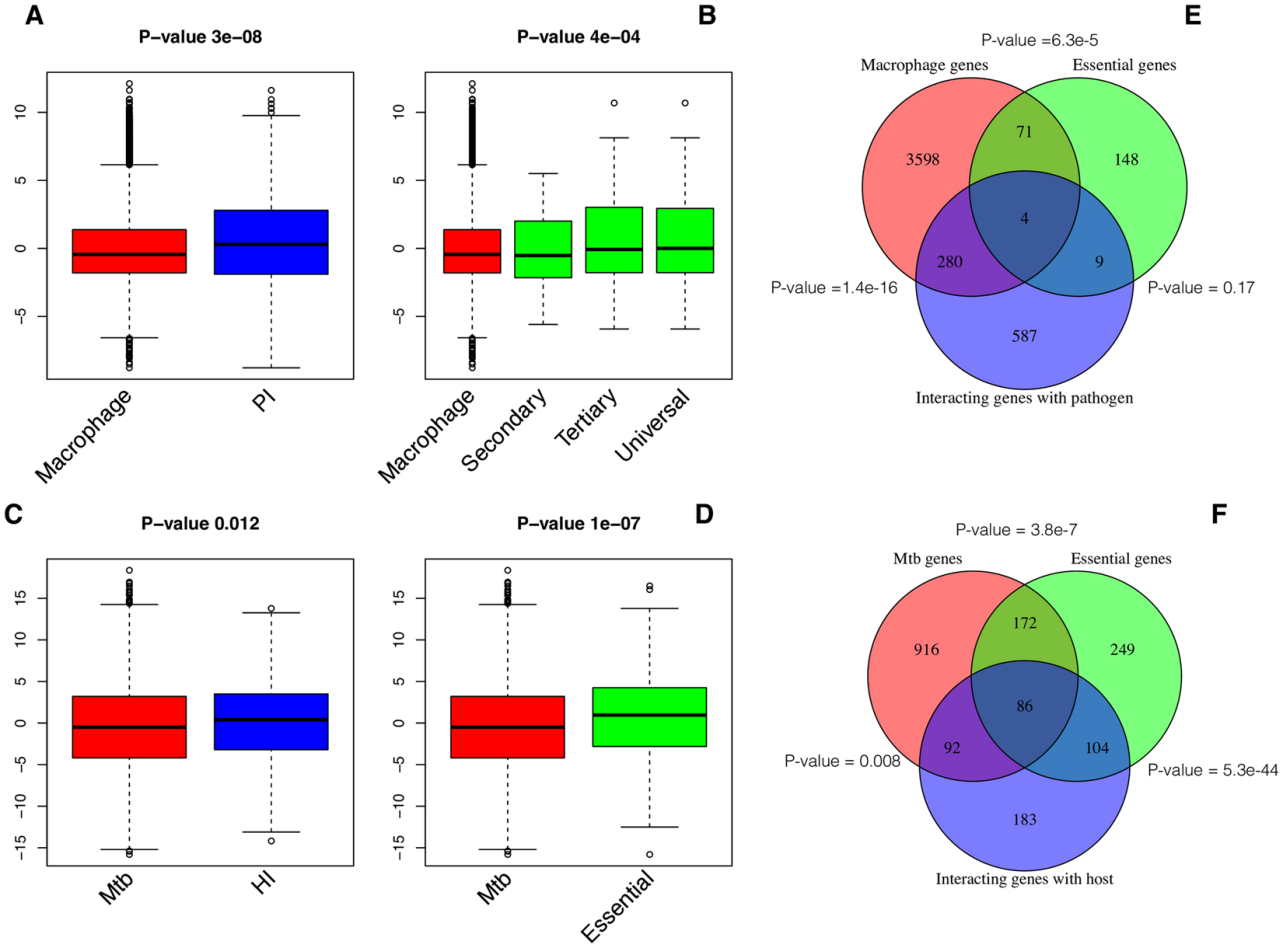
Box plots showing the comparison of differential expression Z-Scores for various gene lists with overall z-scores of the genes induced in the host-pathogen environment. The positive Z-scores indicates up-regulation, whereas negative scores indicate down-regulation; (**A**) Z-scores of total macrophage genes that are differentially expressed upon infection and macrophage genes predicted to be pathogen interacting (PI). (**B**) Z-scores of total macrophage genes that are differentially expressed upon infection and macrophage genes that were reported to be essential to regulate pathogen load using different levels of siRNA screen i.e., secondary, tertiary and universal. (**C**) Z-scores of total *Mtb* genes that are differentially expressed in infected macrophages and *Mtb* genes predicted to be host interacting (HI). (**D**) Z-scores of total *Mtb* genes that are differentially expressed in infected macrophages and *Mtb* genes reported to be essential for survival in different physiological environments. P-value calculated using Wilcoxon rank sum test shows that the distributions of each pair of comparisons are significantly different. (**E**) Venn-diagram with P-value of overlapping statistics between the genes that are expressed with in the macrophages, pathogen interacting and essential (tertiary screen) and (**F**) Venn-diagram with P-value of overlapping statistics between the *Mtb* genes that are expressed with in the macrophages, host interacting and essential.

We identified orthologs of *Mtb* in ~1500 sequenced genomes of bacterial species, which have experimentally verified host-pathogen interactions in HPIDB and PHISTO databases as well as curated from literature^34,35^. The known interactions were then transferred to *Mtb* on the basis of orthology relationships. Totally, we identified 2808 host-pathogen interactions between two sets of proteins 564 from human and 1725 from *Mtb* (Supplementary data S2). Meta-analytic Z-score distribution analysis of meta-analytic ranked list from macrophage and *Mtb* shows that the most of the genes from both sets have above average Z-scores indicating that most of the top ranked genes encode for proteins involved in host-pathogen interactions (Fig. 2A,B). In addition, the studies conducted using genome wide siRNA screen also have large number of genes involved in the regulation of pathogen load and found to have above average Z-scores in meta-analytic ranked list of macrophage genes. (Fig. 2C)^18^. Moreover as the quality of RNAi hits (3 sets of genes) also have above average Z-score in the meta-analytic ranked list, indicating that top ranked genes in the meta-analytic list are highly important for defence against *Mtb*. Similarly, some of the genes reported to be important for survival of *Mtb* in different physiological environments were also have above average Z-scores (Fig. 2D)^32,33,40^. Overlapping statistics using hyper geometric distribution also shows that the top ranked genes from both macrophages as well as *Mtb* were significantly overlap with their respective set of genes that encode for proteins involved in predicted host-pathogen interactions (Fig. 2E,F). There is a significant overlap between a large number of *Mtb* genes that are reported to be essential and the genes, whose products are predicted to interact with the host. The common candidates between the three set *Mtb* genes i.e., essential, host interacting and expressed with in macrophages could be potential targets for therapeutics.

### Higher order functions of *Mtb* genes induced in host-pathogen environment

**Figure 3:**
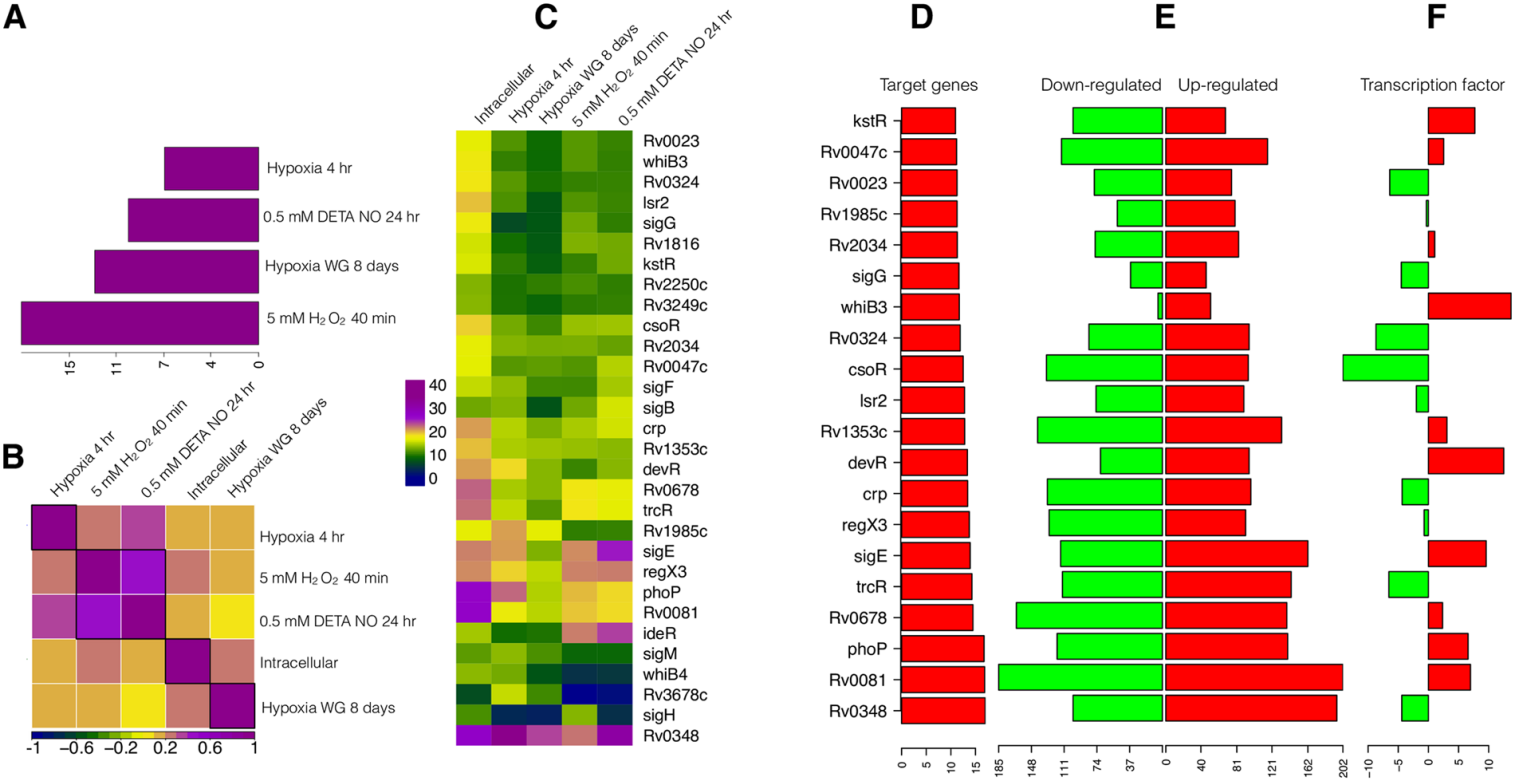
Comparison of meta-analytic ranked list of *Mtb* genes induced in intracellular (macrophage) environment with the genes induced *in-vitro* conditions. (**A**) Bar plot showing the distribution of quantile normalized P-value of Spearman’s rank correlation (threshold value for significance is 2.3) between meta-analytic ranked list with genes induced under different hypoxia and oxidative stress conditions. (**B**) Correlation plot showing the Spearman’s rank correlation (all the correlations have significant P-value) between meta-analytic ranked list with genes induced under different hypoxia and oxidative stress conditions. (**C**) Heat map showing the over/under representation analysis scores for highly differentially expressed target genes of *Mtb* transcription regulators (score >2 is significant) under different conditions. (**D**) Bar plot showing the over/under representation analysis scores for highly differentially expressed target genes of various *Mtb* transcription regulators in intracellular conditions. (**E**) Divergent bar plot showing the number of down-regulated (green bars) and up-regulated genes (red bars) for each of the transcription regulator. (**F**) Bar plot showing the expression level of each of the transcription regulator.

Previously there were many gene expression microarray experiments conducted to identify the adaptive mechanisms of *Mtb* towards the *in-vitro* models of nutrient starvation, hypoxia, and oxidative stress in macrophage environment. There were two types of *in-vitro* models studied under hypoxic conditions, one is constant oxygen (0.2%) supply model and another one is Wayne growth (WG) model, in which the oxygen supply is gradually depleted^41^. In order to identify the nature of environment created by macrophage towards *Mtb* infection, we compared the meta-analytic ranked list of gene expression signatures induced inside macrophages with a large number of previously published gene expression signatures generated using *in-vitro* models of diverse environmental conditions at various time points (GSE16146, GSE8786, and GSE9331)^20,21,42^. The gene expression signatures induced under conditions of constant O_2_ (0.2%) for 4hrs, Wayne growth for 8 days, 5 mM H_2_O_2_ for 40 mins and 0.5 mM DETA (Diethylenetriamine) NO for 24hrs best correlates with the meta-analytic ranked list (intracellular), indicating the likely environmental conditions *Mtb* exposed in macrophage environment (Fig. 3A). Gene expression signatures related to hypoxia model of WG shows stronger correlation than constant O2 (0.2%) supply, model. 5 mM H2O2 shows the strongest correlation. In addition, gene expression signatures induced in all the five different conditions show significant correlation with each other (P-value > 1.5e-08). Particularly, the conditions of hypoxia, H_2_O_2_ and NO show very strong correlation (Fig. 3B).

In order to identify the higher order functions of *Mtb* genes expressed across different conditions and in intracellular environment, we carried out FOA and functional interpretation of regulons, operons and pathways as following^39^.

### Regulons

We curated *Mtb* regulons by collecting known target genes of all transcription factors of *Mtb* from previously published literature^43,44^. We have performed FOA of target genes of various transcription regulators (regulons) in the ranked list of differentially expressed genes across the different conditions to get the list of the transcription regulators, which are critical for adaptation of *Mtb* to macrophage environment. Our analysis shows that the target genes of the persistence regulator, Rv0348 (MosR) were consistently differentially regulated across the different conditions (Fig. 3C) (Supplementary data S3). In addition, the target genes of Rv0081, PhoP, Rv0678, TrcR, SigE, RegX3, Crp, DevR, Rv1353c, Lsr2, CsoR, Rv0324, WhiB3, SigG, Rv2034, Rv1985c, Rv0023, Rv0047c, kstR were also differentially regulated in intracellular environment (Fig. 3D). Although target genes of the RegX3 and WhiB3 were significantly altered in intracellular conditions (Fig. 3E), the respective transcription regulators were not significantly differentially expressed (Fig. 3F). In contrast to the MosR expression, most of its target genes were up-regulated, indicating that it represses the majority of the genes involved in adaptation to the macrophage environment. MosR and its associated genes in an operon *Rv0347-Rv0348-Rv0349* were induced during the late stage of chronic tuberculosis in mice^45^. PhoP is a global regulator of *Mtb* and it plays an important role in pathogenesis and synthesis of cell wall lipids^46^.

### Operons

In most of the bacterial species such as *Mtb*, pathways, complexes and gene ontology terms were not well annotated, which hinders the functional annotation of gene expression signatures. Here, we circumvent the functional annotation problem by inclusion of predicted operons, because genes within the most of operons were known to encode proteins involved in a same pathway or potentially forming a functional protein complex.

**Figure 4:**
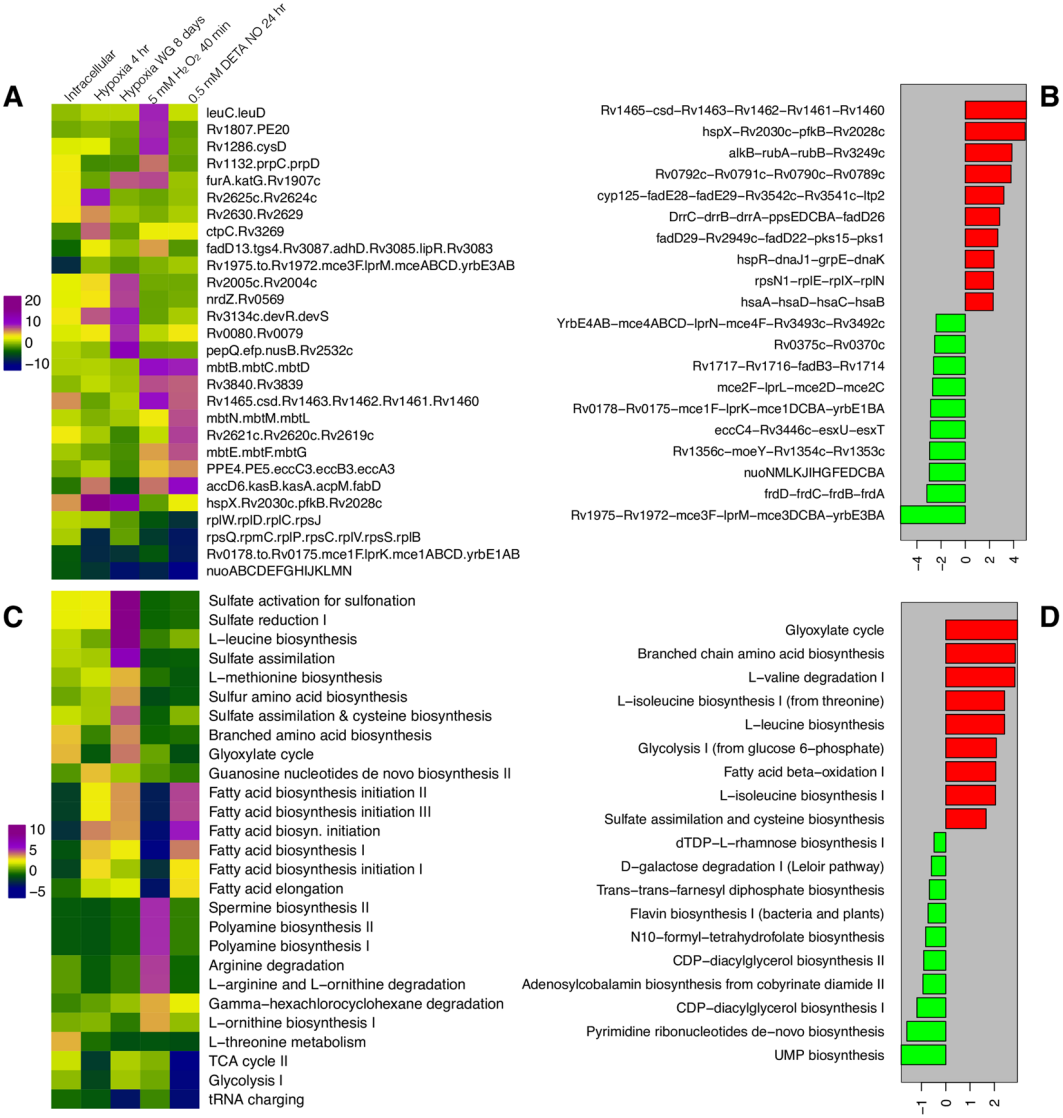
Operons and pathways of *Mtb* with highly differentially expressed genes under different conditions. (**A**) Heat map showing the over/under representation scores of various operons under different stresses and intracellular (macrophage) environment. (**B**) Bar plot showing the operons with highly differentially expressed genes in intracellular environment. (**C**) Heat map showing over/under representation scores of various pathways in different conditions. (**D**) Bar plot showing over/under representation scores of various pathways in intracellular environments.

Over-representation analysis shows that the entire operon encoding for the components of NADH dehydrogenase complex-I were significantly down-regulated across the different conditions of intracellular, hypoxic and oxidative stress (Fig. 4A) (Supplementary data S4), indicating the complete shutdown of oxidative phosphorylation pathway. On the other hand, the operons such as *Rv1465-csd-Rv1463-Rv1462-pps1-Rv1460*, *hspX-Rv2030c-pfkB-Rv2028*, *ceccCa1-eccB1-eccA1-espH-espG1-espF*, *hsaABCD*, *rplNXEN1*, *dnaK-grpE-dnaJ1-hspR*, *pks1-pks15-fad22-fad29*, *fadD26-ppsABCDE-drrABC*, *ltp2-fadE29-fadE28-cyp125* were highly expressed in intracellular conditions (Fig. 4B). The pps1 locus constitutes an operon with seven genes (*Rv1465-Rv1460*), which code for Suf-like proteins, which are part of SUF machinery, an exclusive Fe-S cluster assembly existing in *Mtb*^47^. The Pps1 protein is orthologous to SufB which is essential and conserved component of iron sulphur cluster assembly (Fe-S cluster) complex of prokaryotes. It was reported that as Fe-S clusters serve as cofactors of proteins which are involved in multiple cellular mechanisms^48^.

The operon *hspX-Rv2030c-pfkB-Rv2028* was reported to be essential for the *Mtb* during persistent infection in the host and also for its survival during low iron, low pH and hypoxic condition^49–51^. The protein encoded by *hspX* gene of the operon is known to be highly up-regulated during the latent infection and reported to play an important role in anaerobic respiration of *Mtb* during stress conditions. The other genes of the operon are found to be involved in carbohydrate metabolism (*pfkB*) and nucleotide metabolism (*Rv2030c*)^49–51^.

The two operons *hsaABCD* and *cyp125-fadE28-fadE29-Rv3542c-Rv3541c-ltp2* were highly conserved across the members of *Mtb* complex^52,53^. They participate in cholesterol degradation pathway and play an important role in intracellular survival of *Mtb* by providing cholesterol as an alternative carbon source^54–59^. Another two operons *pks1-pks15-fad22-fad29* and *fadD26-ppsABCDE-drrABC* were involved in synthesis of phthiodiolone dimycocerosates (DIMs) and immuno-modulatory phenolic glycolipids, which have a role in immuno-modulation^60–62^. Some of the genes (*drrA*, *drrB* and *drrC*) in aforementioned operon were known to encode for a drug efflux pump, which confers resistance to many antibiotics^63^. The genes with in the operon (*alkB-rubA-rubB-Rv3249c*) encode for alpha-ketoglutarate-dependent dioxygenase (*alkB*), which catalyses oxygenation of hydrocarbons^64^, could be having role in cholesterol degradation. The genes involved in cholesterol utilization and other genes (*Rv0792c*, *Rv0791c*, *Rv0790c*, *Rv0789c*) with unknown function were highly expressed in intracellular environment in comparison to the other conditions.

Comparative analysis of operons with the genes induced across different conditions has identified several functions that are common and unique to each of the environment. Pathways involved in biosynthesis of iron-sulfur cluster, mycobactins (*mbtB-mbtC-mbtD*) and meromycolic acids (*accD6*-*kasB*-*kasA*-*acpB*-*fabD*) were highly expressed in presence of NO and H_2_O_2_ indicating their role in oxidative stress defence_._ On the other hand the genes with in the operons (*devR*-*devS*-*Rv3134c* and *hspX*-*Rv2030c*-*pfkB*-*Rv2028c*) were highly expressed during hypoxic conditions. Furthermore the genes with in the operons (*furA*-*katG*-*Rv1907c* and *ahpC-ahpD*) were expressed across intracellular, hypoxic, H_2_O_2_ induced oxidative stress conditions. In contrast to the previous reports, co-expression of *katG* and *furA* indicates that *furA* may not be a repressor of *katG* under these conditions^65^.

### Pathways

Over-representation analysis of various *Mtb* pathways among the genes induced during infection shows that the glyoxylate pathway which is known to play an important role in persistence of *Mtb* in macrophages^66^ is only active in Wane growth model of hypoxia and intracellular conditions (Fig. 4C) (Supplementary data S4). In addition, pathways involved in the biosynthesis of chorismate and branched chain amino acids such as L-leucine, L-isoleucine (from threonine) and assimilation of sulfate and cysteine biosynthesis were highly expressed during hypoxia and intracellular conditions, but not during oxidative stress. Furthermore, various catabolic pathways such as fatty acid & beta-oxidation I, glycolysis I (from glucose 6-phosphate), and glyoxylate cycles are highly expressed during intracellular conditions (Fig. 4D). The chorismate pathway, also called as shikimate pathway is found in *Mtb* but absent in humans and other mammals^67,68^. This pathway produces chorismate, which is a key intermediate compound for the biosynthesis of amino acids, ubiquinones, siderophores and other important metabolites essential for *Mtb* growth and survival in the host^69–73^. Overall, the pathways involved in the biosynthesis of L-Leucine, chorismate, cysteine, and assimilation of sulfate are linked to the long-time survival of *Mtb* during the pathogenesis.

### Higher order functions of differentially expressed macrophages genes upon *Mtb* infection

#### Pathways

**Figure 5:**
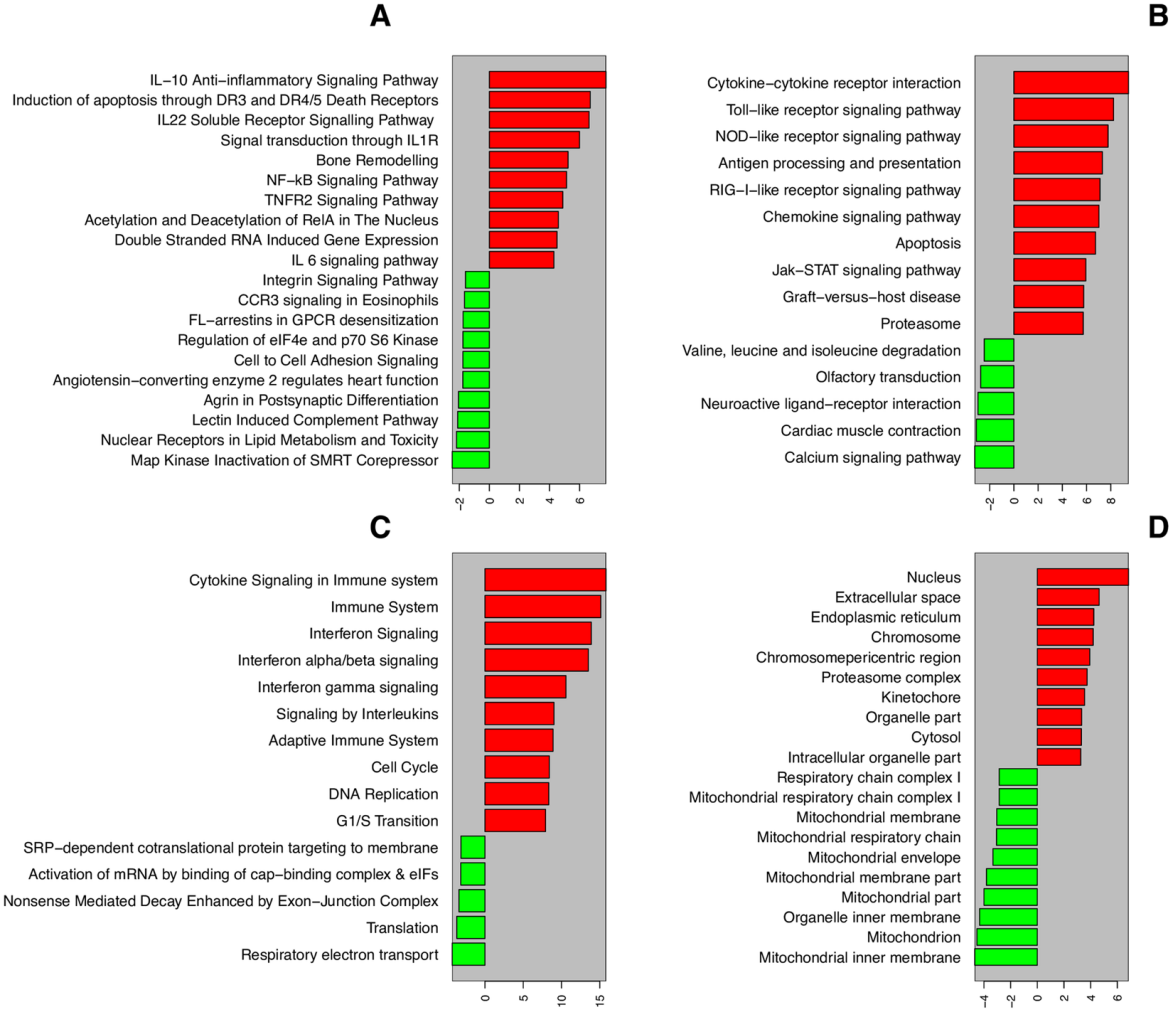
Macrophage pathways and cellular components with highly differentially expressed genes upon infection. (**A**,**B** and **C**) shows over/under represented pathways from Biocarta, KEGG and Reactome, respectively. (**D**) shows the over/under represented cellular components.

In order to identify the macrophage pathways that were induced during infection, we have carried out over-representation analysis of pathway genes from various databases such as BioCarta, KEGG, and Reactome^74–76^ (Fig. 5) (Supplementary data S5). The BioCarta pathways namely, IL-10 anti-inflammatory signalling, induction of apoptosis through DR3 and DR4/5 Death Receptors, IL22 soluble receptor signalling, NF-kB, TNFR2 signalling and Acetylation/deacetlyation of RelA were found to be highly expressed (Fig. 5A).

IL-10 family of cytokines has a total of 6 IL’s. In contrast to the rest of the IL-10 family of cytokines, IL-10 acts as an anti-inflammatory cytokine and is also capable of inhibiting the synthesis of pro-inflammatory cytokines such as IL-2, TNF-α, IL-3, IFN-α which indirectly leads to the survival of *Mtb* inside the infected macrophage. Supporting this fact, there are reported evidence from the infection studies of *Mycobacterium avium sub species paratuberclosis* (MAP). It was documented that MAP adopts a different strategy to escape from the host defence. MAP activates the mitogen activated protein kinase pathway, which finally results in up-regulation of IL-10 and repression of other inflammatory cytokines. Further the up-regulated IL-10 also inhibits the apoptosis in macrophages^6,77,78^. According to our findings and the supporting evidence, it may be assumed that the up-regulation of IL-10, an anti-inflammatory pathway of macrophage may aid in the growth and virulence of *Mtb* and may also be considered as one of the immune-modulatory strategies adopted my *Mtb* to escape from host defence mechanisms for its survival.

The pathway ‘induction of apoptosis through the engagement of death receptors’ has a role in apoptosis of infected macrophages via engagement of variety of death receptors such as Fas, TNFR, DR3, DR4 and DR5^79^. Apoptosis of this kind, where there is engagement of death receptors is the sign of innate immune response of macrophages against *Mtb*^79^. IL22 soluble receptor signalling pathway was reported to inhibit the growth of *Mtb* by enhancing the expression of intracellular signalling molecule, calgranulin A. In addition, it was reported to be involved in tissue modulation, induction of proliferative and anti-apoptotic pathways and production of anti-microbial substances^80^.

NF-kB pathway, inflammatory cytokine and TNF-α are responsible for inflammatory response towards *Mtb* infection^81^. TNF- α shows its immune response in variety of ways such as macrophage activation, induction of cytokines, chemokines and induction of apoptosis and also restriction of bacterial growth in infected macrophage^81–85^. TNFR2 signalling pathway is mainly responsible for apoptosis, acetylation/deacetlyation of RelA and is responsible for innate immune response against *Mtb*. Overall the over-representation analysis in Biocarta pathway shows that all the pathways related to recognition of molecular patterns of *Mtb* are responsible for triggering inflammation and apoptosis, are up-regulated.

Over-representation of analysis of KEGG pathways show that the genes involved in cytokine-cytokine receptor interaction, toll-like receptor signalling, NOD-like receptor signalling, RIG-I like signalling, chemokine signalling and apoptosis signalling were highly up-regulated in the macrophages during *Mtb* infection (Fig. 5B). After infection of *Mtb* in pulmonary alveoli, macrophages express pattern recognition receptors (PRR) which in turn recognize the pathogen associated molecular patterns (PAMPS). The recognition of PAMPS by PRRs leads to the activation of transcription factors, which are involved in providing innate immunity^86^. In the case of mycobacterial infections, mycobacterial cell wall-associated components such as lipoarabinomannan are recognized by TLR1 and TLR2. Similarly there are several cell wall associated components which may be recognized during the time of mycobacterial infection as reported earlier^87–89^. In NOD-like receptor signalling pathway, the NOD like receptors belonging to NLR family and they may play key roles in innate immunity and inflammation^90^. They recognize the peptidoglycan fragments of the pathogens escaped from endosomal compartments leading to the activation of cytokine production and ultimately resulting in programmed cell death^91,92^.

Apart from the NOD like receptors, there are also few other proteins called as cytosolic RNA helicases (RIG-I like signalling pathway) that were also highly up-regulated. These cytosolic RNA helicases (RIG-I-like receptors) are reported to recognize the antigens from viral pathogens and induce the innate immune response against them by triggering the signalling pathways which produce the type I interferon and inflammatory cytokines^93,94^. These have been reported to be involved in the intracellular detection of all pathogens including *Mtb*^95^. Chemokine signalling pathway plays an important role in migration of leukocytes to the site of infection and orchestrates the host defence mechanism^96^.

The genes involved in apoptotic pathways were highly up-regulated (Fig. 5A,B). The cells follow two different kinds of pathways for undergoing apoptosis: the death receptor or extrinsic pathway and the mitochondrial or intrinsic pathway, where the final dead cells are cleared through phagocytosis by the macrophages^97–99^. In addition, the pathways in involved in branched chain amino-acid degradation were down-regulated, unlike in intracellular *Mtb*, in which the biosynthetic pathways of branched chain amino-acids were up-regulated as mentioned previously.

Analysis of Reactome pathways show that there are two kinds of the pathways that were up-regulated during infection: pathways pertaining to immune system activation and pathways responsible for cell survival and growth. The former category consists of cytokine signalling related genes, interferon signalling, interleukins signalling and adaptive immunity. The second category consisting of the pathways related to cell cycle, DNA replication and G1/S transition pathways were also observed to be highly up-regulated (Fig. 5C). In the first category, most of the pathways which were highly regulated were found to be cytokines with diverse functions. As previously described, cytokines have immune response triggering properties against the infections in the host. Interferon’s are also cytokines (pleiotropic cytokines) which have immune-regulatory properties 103. In our analysis, we also have found the pathways pertaining to interferon α/β/γ signalling as highly up-regulated which are also actively involved in triggering immune response. These results confirm that the pathways in the first category are involved in providing innate and adaptive immunity against infection. The pathways which are involved in cell cycle, DNA replication, G1/S transition are well documented to be linked with cell growth and survival.

#### Cellular components

Over-representation analysis of genes encoding various cellular components shows that the genes that encode for proteins localized to Endoplasmic Reticulum (ER) were observed to be up-regulated during infection. In macrophages, the mycobacterial antigen ESAT-6 evokes the ER stress, which in turn mediates the process of apoptosis^100^. This induction of apoptosis further leads to the killing of *Mtb*. The other highly overrepresented proteins in the cellular localization data set are mainly related to the intracellular proteins associated with kinetochore (Fig. 5D) (Supplementary data S5). In contrast to the ER, most of the mitochondrial components were down-regulated, indicating the shutdown of most of the mitochondrial functions in infected macrophages. *Mtb* virulence is known to correlate with mitochondrial cytochrome c release in infected macrophages^101^. Mitochondria could be an important target for mycobacteria, because of its involvement in the regulation of immune response and cell survival/programmed cell death^102–104^. Furthermore, the *Mtb* could also be exploiting some of the mitochondrial functions considering that mitochondria derived from endosymbiosis of an ancestral bacteria^105^. It has been shown that mycobacterial proteins carry mitochondrial localization signals, which may help its virulence proteins to be transported to the mitochondria and further hijack it’s functions^106^.

## Conclusions

In this report by performing the meta-analysis of publicly available gene expression datasets and analysis of higher order functions and interactions, we have derived comprehensive view about cross talk between *Mtb* and macrophages during the stable infection. We observed a strong correlation of gene expression signatures between the intracellular bacteria with *in-vitro* models of dormancy conditions, which reflects the likely environmental conditions in existing in macrophages at the time of infection. Moreover from this analysis, it is clear that *Mtb* effectively fights against the oxidative, starvation and hypoxic conditions presented by the host as defence against infection. Analysis of different functional signatures associated with different stress conditions provides the valuable information related to the strategies adapted by *Mtb* inside the macrophage for its survival and persistence. For instance such as, the mechanisms adapted by *Mtb* to encounter the oxidative stress induced by intracellular H_2_O_2_, through catalase-peroxidase and alkyl hydro peroxide reductase, similarly, recruitment of iron-sulfur clusters, mycobactins and meromycolic acids to encounter the oxidative stress induced by NO shows the strategies adapted by *Mtb* to survive in infected macrophage. Further, from our analysis it is observed that, *Mtb* effectively tackles the harsh environmental conditions of macrophage by intracellular environment by up-regulation of cholesterol utilization pathways and bio-synthesis of immuno-modulatory phenolic glycolipids. Further study of these pathways and other genes with unknown function would establish their role and mechanisms of action to counter each of the stress conditions posed by macrophage environment. In addition possible pathways of *Mtb* that target and exploit mitochondria should be identified. This study clearly inferred that, when therapeutic strategies to combat dormant *Mtb* should be designed, the multiple pathways involved in adaptation to different stress conditions and pathways that target mitochondrial functions should be taken into consideration.

## Electronic supplementary material

Supplementary data 1

Supplementary data 2

Supplementary data 3

Supplementary data 4

Supplementary data 5
